# Circulation of Indigenous Bovine Respiratory Syncytial Virus Strains in Turkish Cattle: The First Isolation and Molecular Characterization

**DOI:** 10.3390/ani10091700

**Published:** 2020-09-20

**Authors:** Zafer Yazici, Emre Ozan, Cuneyt Tamer, Bahadir Muftuoglu, Gerald Barry, Hanne Nur Kurucay, Ahmed Eisa Elhag, Abdurrahman Anil Cagirgan, Semra Gumusova, Harun Albayrak

**Affiliations:** 1 Department of Veterinary Virology, Faculty of Veterinary Medicine, Ondokuz Mayis University, 55139 Samsun, Turkey; cuneyt_tamer@hotmail.com (C.T.); kurucayhannenur@gmail.com (H.N.K.); semragumusova@hotmail.com (S.G.); harunalbayrak55@msn.com (H.A.); 2 Department of Veterinary Experimental Animals, Faculty of Veterinary Medicine, Ondokuz Mayis University, 55139 Samsun, Turkey; emre.ozan@omu.edu.tr (E.O.); bahadirmuftuoglu@hotmail.com (B.M.); 3 Veterinary Science Centre, School of Veterinary Medicine, University College of Dublin, Dublin 4, Ireland; gerald.barry@ucd.ie; 4 Bornova Veterinary Control Institute, Veterinary Control Institute Directorates, Ministry of Agriculture and Forestry, 35010 Izmir, Turkey; a.anilcagirgan@gmail.com

**Keywords:** BRSV, cattle, isolation, respiratory disorders, sequencing

## Abstract

**Simple Summary:**

Bovine respiratory syncytial virus (BRSV) is an important pathogen of both dairy and beef cattle, and causes huge economic losses annually across the world. This study reports the identification, isolation, and molecular characterization of a new BRSV (subgroup III) strain collected from respiratory distressed cattle in Turkey. The three field isolates obtained showed 100% similarity to each other at the nucleotide (nt) level and were found to be 99.49% and 99.22% identical to another Turkish strain—KY499619—at both (nt) and amino acid (aa) levels, respectively. They were also 97.43% (nt) and 98.44% (aa) similar to the American reference strain KU159366. This important information will inform Turkish BRSV diagnostic and control strategies, as well as highlight the urgent need to better understand the burden that BRSV is placing on the Turkish agricultural sector.

**Abstract:**

Bovine respiratory disease (BRD) is a huge economic burden on the livestock industries of countries worldwide. Bovine respiratory syncytial virus (BRSV) is one of the most important pathogens that contributes to BRD. In this study, we report the identification and first isolation, with molecular characterization, of a new BRSV strain from lung specimens of three beef cows in Turkey that died from respiratory distress. After the screening of lung tissues for BRD-associated viruses using a multiscreen antigen-ELISA, a BRSV antigen was detected. This was then confirmed by real-time RT-PCR specific for BRSV. Following confirmation, virus isolation was conducted in MDBK cell cultures and clear CPE, including syncytia compatible with BRSV, were detected. RT-nested PCR, using F gene-specific primers, was performed on the cultured isolates, and the products were sequenced and deposited to Genbank with accession numbers MT179304, MT024766, and MT0244767. Phylogenetic analysis of these sequences indicated that the cattle were infected with BRSV from subgroup III and were closely related to previously identified American and Turkish strains, but contained some amino acid and nucleotide differences. This research paves the way for further studies on the molecular characteristics of natural BRSV isolates, including full genome analysis and disease pathogenesis, and also contributes to the development of robust national strategies against this virus.

## 1. Introduction

Bovine respiratory disease (BRD) is a term used to describe respiratory disease in cattle caused by single or a range of pathogens. It is a complex disease, with multiple viruses, bacteria, and parasites potentially involved [1,2]. BRD can negatively affect production and is therefore considered a major economic burden on the livestock industry worldwide [1,3]. Bovine respiratory syncytial virus (BRSV), also called *Bovine orthopneumovirus,* is an important pathogen of this complex [2,4,5]. BRSV belongs to the genus *Orthopneumovirus* of the family *Pneumoviridae* in the order *Mononegavirales* [6] and has a negative sense, single-stranded RNA genome. The non-segmented virus genome consists of 10 genes that encode 11 proteins: Two non-structural proteins (NS1 and NS2); a nucleocapsid (N) protein; a phosphoprotein (P); a matrix protein (M); glycoproteins SH, G (attachment), and F (fusion); M2-1 and M2-2 (control transcription and RNA replication); and RNA polymerase (L) [7,8].

Phylogenetic analysis based on both F and G proteins has led to a subdivision of BRSV into eight subgroups, denoted I–VIII [7,9,10,11]. These subgroups tend to separate geographically; subgroup I BRSV strains are typically isolated in the UK and Switzerland, whereas subgroup II normally includes strains from the Netherlands, Denmark, Belgium, France, and Japan [9,12]. The strains from the USA are commonly included in subgroup III, while some strains from the USA and other European countries fall into subgroup IV [7,9]. Finally subgroups V and VI include BRSV strains usually found in Belgium and France [9]. Subgroups VII and VIII are a recent addition and are predominantly seen in Europe, particularly in Italy and Croatia [7,10]. In addition, a putative new BRSV subgroup, tentatively named subgroup IX, has been proposed, in order to classify recent strains isolated from Brazil, which have mutations in the immunodominant region of the G protein [11].

BRSV predominantly infects cattle, although sheep and goats can also be infected [8,12]. The virus is mainly transmitted by direct contact and/or aerosols and both clinical and sub-clinical animals are capable of transmitting [7,13,14]. BRSV outbreaks can occur in all ages of animals, but young calves (particularly those between 2 weeks and 9 months old) are especially vulnerable [12,13].

The clinical manifestations of BRSV infections in cattle can vary from mildly symptomatic to fatal, and the outcome is multifactorial. The breed of cattle; the strain of virus; and the contribution of other viruses, bacteria, and parasites all play a role, alongside other factors, such as management practices and environments [5,14,15]. Although BRSV infection is rarely fatal, it can lead to upper and lower respiratory damage in young calves and is characterized by a fever, cough, decreased feed intake, increased respiratory rate, and nasal discharge [14,15].

There is limited information available about the seroprevalence of BRSV in Turkey and very few BRSV sequences from Turkish isolates have been submitted to GenBank [4,5]. Cases of BRD are plentiful in Turkey and some have been characterized, but major gaps in our understanding remain, thus limiting the ability to control it [3,16]. In this context, the aim of this study was to present both the first isolation of BRSV in Turkey and its molecular characterization, consisting of both sequencing and phylogenetic analysis.

## 2. Material and Methods

### 2.1. Samples

Between December 2018 and January 2019, a veterinarian in the Samsun province of Northern Turkey reported three cases of respiratory disease in beef cattle from the same farm. The cattle were unvaccinated against any BRD-associated viruses, including BRSV. The cattle died from severe respiratory disorders, including pneumonia. Lung tissue samples were taken post-mortem and sent to the Virology Department of The Faculty of Veterinary Medicine, Ondokuz Mayis University, for diagnosis. To differentiate the samples, they were labeled 34TR2018, 43TR2018, and 07TR2019, according to the animal they came from.

### 2.2. Virus Isolation

For cell culture isolation, approximately 1 g of lung tissue was placed in 5 mL of cold Minimal Essential Medium (MEM) containing 2% penicillin/streptomycin (Sigma-Aldrich, St. Louis, MO, USA), and then homogenized on ice for 1 min at 6000 rpm using a tissue homogenizer (Heidolph Ins., Schwabach, Germany). The homogenate was then centrifuged at 1500× g for 15 min and the supernatant was sterile-filtered (0.22 μm) and stored at −20 °C. For PCR, approximately 30 mg of lung tissue was homogenized with a Tissue-Lyser (Qiagen AG, Hilden, Germany) in 1.8 mL of MEM containing 2% penicillin/streptomycin. Obtained homogenates were clarified by centrifugation at 1500× g for 15 min and the supernatant was then sterile-filtered (0.22 μm) and stored at −20 °C.

MDBK cells were used for the virus isolation studies. Briefly, MDBK cells were cultivated at 37 °C with 5% CO_2_ in Dulbecco’s Modified Eagle’s Medium (DMEM, Gibco, Paisley, UK) supplemented with 10% fetal calf serum (FCS, Sigma-Aldrich) and 1% penicillin/streptomycin (Sigma-Aldrich). Virus isolation from suspected samples was conducted by performing blind passages. Supernatants from tissue homogenates were inoculated onto MDBK monolayers at 37 °C for 60 min and then replaced with DMEM containing 2% FCS. Cells were maintained at 37 °C with 5% CO_2_, and checked daily for cytopathic effects (CPE).

### 2.3. Antigen-ELISA for BRD-Associated Viruses

The lung tissues were tested using a commercially available multiscreen antigen-ELISA kit (Bio-X, Rochefort, Belgium, Cat. No: BIO K, 340/5). The kit has been reported to detect Bovine parainfluenza-3 (BPIV-3), Bovine viral diarrhea virus (BVDV), BRSV, and Bovine herpesvirus-1 (BHV-1), and was used according to the manufacturer’s instructions.

### 2.4. Nucleic Acid Extraction and Amplification

We performed RNA extractions from homogenized lung tissue, as well as infected cell culture lysates, using a GeneJET RNA Purification Kit (Thermo Fisher Scientific, Vilnius, Lithuania), according to the manufacturer’s instructions. The extracted RNA was eluted in 75 µL of elution buffer and kept at −80 °C.

For the real time RT-PCR analysis, the primers and the TaqMan probe targeted the N gene of BRSV and have been previously described by Boxus et al. [17]. For the RT-nested PCR, F gene-specific primers were used as previously described by Vilcek et al. [18]. For the first round of RT-nested PCR, we used B1 and B2A primers that amplify a 711 bp product. This was followed by B3 and B4A primers that amplify a 481 bp product. The sequence data of the primers and probe are given in Table 1.

The real time RT-PCR was carried out using an iTaq™ Universal Probes One-Step Kit (Biorad, Hercules, CA, USA, Cat No: 1725140) on a CFX Connect real time PCR machine (Biorad). The real time RT-PCR reactions were carried out in a final volume of 25 μL containing 5 μL of RNA, 12.5 μL of 2X buffer, 320 nM of each primer, 160 nM of probe, 0.5 μL of RT enzyme, and 5 μL of RNAse-free water. The PCR conditions were as follows: 10 min at 50 °C for reverse transcription and 3 min at 95 °C, followed by 45 cycles at 95 °C for 7 s and 59 °C for 10 s.

The RT-nested PCR was carried out using the Qiagen Onestep RT-PCR kit (Qiagen, Cat No:210212). The first round of PCR was performed in a final volume of 50 μL consisting of 5 μL of RNA, 10 μL of 5X buffer, 400 nM of each primer, 1 μL of dNTP, 1 μL of RT enzyme, and 29 μL of RNAse-free water. The PCR conditions were as follows: 30 min at 50 °C for reverse transcription and 15 min at 95 °C, followed by 35 cycles at 95 °C for 45 s, 50 °C for 45 s, 72 °C for 60 s, and finally a cycle at 72 °C for 10 min. The second round of PCR was also carried out in a final volume of 50 μL containing 5 μL of the first round RT-PCR product, 10 μL of 5X buffer, 400 nM of each primer, 1 μL of dNTP, 1 μL of RT enzyme, and 29 μL of RNAse-free water. The cycling conditions employed for the second round of PCR were as follows: 1 min at 95 °C and 45 s at 95 °C, followed by 35 cycles at 50 °C for 45 s, 72 °C for 1 min, and finally a cycle at 72 °C for 10 min. Positive controls for both RT-PCR tests were provided by the virology laboratory of the Samsun Veterinary Control Institute, Turkey. For the nested PCR, all amplicons were visualized on 1% agarose gels.

### 2.5. Sequencing and Phylogenetic Analysis

PCR products were purified using a QIAquick PCR purification kit (Qiagen), according to the manufacturer’s instructions and then Sanger sequenced by RefGen Biotechnology, Ankara, Turkey (http://www.refgen.com). The sequences were aligned using Bioedit, version 7.2.5, followed by BLAST analysis in GenBank databases [19]. For comparison, we selected seventeen representative isolate sequences from GenBank, including BRSV and human respiratory syncytial virus (HRSV) strains. The phylogenetic tree was constructed with the maximum likelihood method under the Tamura-3 parameter model using MEGA X (Molecular Evolutionary Genetics Analysis-MEGA, version 10.0.5) [20], and the bootstrap values were based on F gene nucleotide (nt) sequences. The tree was assessed using 1000 bootstrap replications. The sequences identified from animals 07TR2019, 34TR2018, and 43TR2018 were deposited in GenBank with the accession numbers MT179304, MT024766, and MT0244767, respectively.

## 3. Results

### 3.1. The Identification of Lung Tissue Samples Infected with BRSV

The first step of this study was the screening of all lung tissues for BRD-associated viruses using both the multiscreen antigen-ELISA and real time RT-PCR. All tissue samples were BRSV positive by ELISA, and negative for BVDV, BHV-1, and BPIV-3. The samples were also confirmed to be positive for BRSV by real time RT-PCR.

### 3.2. Virus Isolation

**Figure 1:**
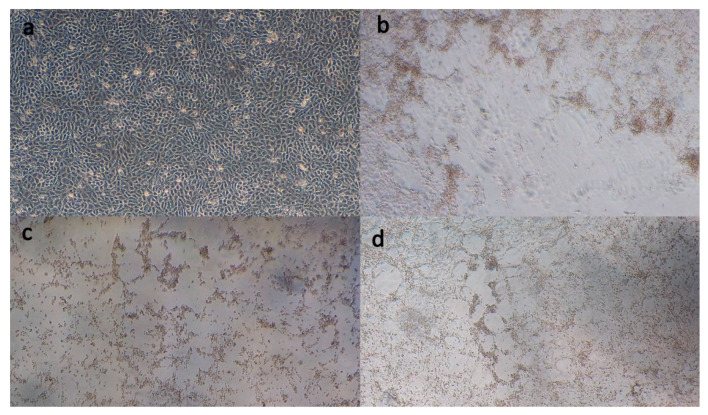
Photos of MDBK cells: (**a**) Cell control; (**b**,**c**) MDBK cells displaying cytopathic effects (CPE) and syncytia formation 72 h post-infection with isolates 43TR2018 and 34TR2018, respectively; (**d**) MDBK cells displaying limited CPE and syncytia foci 72 h post-infection with isolate 07TR2019.

Lung homogenates from each animal, consisting of 43TR2018, 342TR2018, and 07TR2019, were added to MDBK cultures and incubated. Obvious CPE including syncytia were visible between 3- and 4-days post inoculation (Figure 1).

### 3.3. RT-Nested PCR

Following virus propagation in culture, RNA was extracted from each of the infected MDBK cultures and nested PCR was performed. Based on this, 711 and 481 bp bands corresponding to the F gene were visible for 43TR2018 and 07TR2019, while only a 481 bp band, corresponding to the second round of PCR, was visible for isolate 34TR2018.

### 3.4. Sequencing and Phylogenetic Analysis

Following the RT-nested PCR, the 481 bp fragments were sequenced. As detailed in Table 2, the sequencing showed that the three isolates—MT179304, MT024766, and MT024767—had a 100% similarity to each other at the nucleotide (nt) level. Furthermore, the three isolates were found to be 99.49% and 99.22% identical to another Turkish strain—KY499619 [3]—at both nt and amino acid (aa) levels, respectively. The three isolates presented here were also 97.43% (nt) and 98.44% (aa) identical to the American reference strain KU159366.

**Figure 2:**
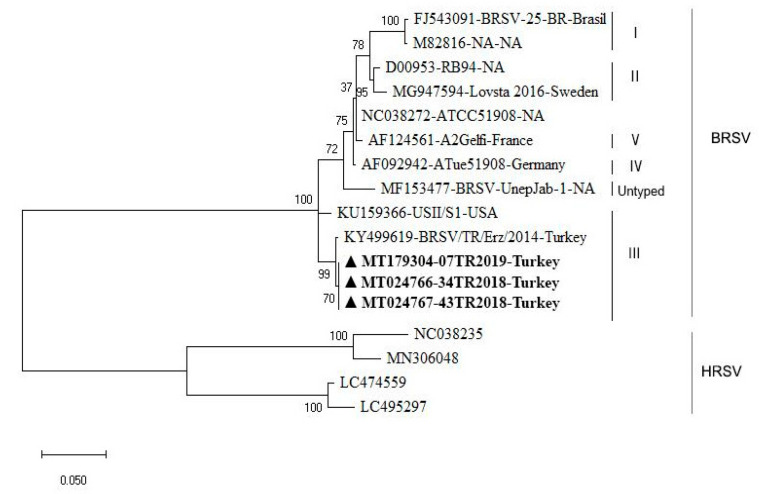
The phylogenetic analysis of the BRSV strains isolated in this study. The tree was constructed from a partial analysis of the BRSV F gene using the maximum likelihood method with MEGA X software. The robustness branching pattern was tested with 1000 bootstrap replications. According to the phylogenetic tree, the current strains were in subgroup III (marked in bold and with a ▲). The BRSV sequences were named using their GenBank accession number, strain name, and geographical origin. NA: Not available.

As depicted in Figure 2, the phylogenetic analysis of the F gene revealed that our isolates—MT179304, MT024766, and MT024767—are in the same cluster in subgroup III, together with isolates KY499619 and KU159366, based on the nucleotide sequence.

Interestingly, however, there are some small amino acid differences that exist between these newly presented isolates and KY499619 and KU159366 (Table 3). Unlike the three isolates presented here, in isolate KU159366, a Threonine (T) at position 118 is an Alanine (A) and a Threonine (T) at position 173 is a Serine (S), while a Lysine (K) at position 176 is a Glutamic Acid (E) in isolate KY499619.

## 4. Discussion

BRD is an economically important condition in the worldwide livestock industry that can be caused by a number of different viruses, bacteria, and parasites, individually or in combination [2,21]. The disease is typically caused by early virus infection, followed by bacterial secondary infection that can lead to lung damage and pneumonia-like illnesses [2,21]. Surprisingly, we have recently observed cattle with severe, sometimes fatal, BRD, associated with just single-pathogen infections, such as BRSV, BPIV3, or BHV-1 [3,5,22].

To investigate this and to understand potential changes in the viruses that may be causing an increased virulence, it is vital to be able to isolate and study the viruses in question. Previous studies in Turkey have mainly been diagnostic in nature, with limited phylogenetic analysis [4,5,23,24]. We present here, for the first time, the isolation and growth in culture of BRSV from infected cattle, phylogenetic analysis of the aforementioned isolates, and the identification of differences between them and previous isolates from Turkey and abroad. In this study, lung tissues from diseased animals were collected post-mortem and screened for the most common BRD-associated viruses, including BVDV, BHV-1, BPIV3, and BRSV, using commercially available ELISA kits, and only a BRSV antigen was found to be present [5]. No screening for bovine coronavirus or bacteria in the lung tissue was carried out, so the contribution from these pathogens to the disease state of the animals cannot be ruled out. Following a positive ELISA, lung homogenates were added to the cell culture and observed daily until CPE including syncytia and round apoptotic cells were visible. The isolation of BRSV in culture can be challenging because the virus is relatively labile and can struggle to grow in culture; however, this successful isolation now opens the door to extensive studies that would not have been possible otherwise [25,26,27].

The F gene of BRSV encodes a major structural protein that is commonly targeted by the adaptive immune response. It is a protein that is central to virus entry into cells, as well as being responsible for the fusion of infected cells with adjacent cells, resulting in the formation of large multinucleated syncytia [28]. Furthermore, the F gene is also a highly conserved region of the BRSV genome compared with the G gene [11]. The results of partial sequencing of the F gene of the current three isolates revealed that they were 100% identical to each other and were closely related to the sequence submitted (KY499619) from Turkey by Timurkan et al. [3]. We determined that the current strains—MT179304, MT024766, and MT024767—had a 99.49% nt and 99.22% aa similarity to the KY499619 strain. When compared to an international strain—KU159366—the isolates had a 97.43% nt and 98.44% aa similarity.

Phylogenetic analysis classified the isolates in subgroup III, similar to isolate KY499619, which was previously identified in Turkey and KU159366 from the USA. Considering the geographical location of Turkey, one could imagine a scenario where European strains would be readily imported into Turkey as globalization and international trade may hold potential risks for the spreading of diseases [29]; however, this does not seem to be the case for the outbreak on the farm in the present study. The amino acid change at position 118 is particularly interesting because that is the −2 position for an N-glycosylation sequence. The −2 position can have an influence on the efficiency of glycosylation of the local Asparagine [30]. Position 118 is also in the middle of the virokinin that is cleaved from the fusion protein of BRSV and is known to increase pulmonary inflammation during infection [31,32]. It is possible that this glycosylation efficiency may impact cleavage at the fusion protein furin cleavage sites, or that the amino acid change may impact the function of the virokinin itself. The second amino acid difference identified in the fusion protein compared to strain KY499619 is in the F2 subunit. Changes in this region could impact antibody recognition and may have consequences for vaccine development. All of these questions are areas for future exploration using the isolates in culture from this study.

Turkey has strong economic links to the USA and live animals are transported to Turkey from this region [33]. This is a possible source of the virus and illustrates the risks associated with globalized trade. Animals are known to harbor BRSV without symptoms, meaning the virus could easily be transported without detection [3], and constant introductions of new strains will increase the diversity of viruses in circulation, thus potentially complicating control efforts.

While the sample number is small in this study and from just one farm, it is a clear warning to the agricultural industry in Turkey that variant strains of BRSV exist in Turkey—variants that may have an increased virulence—and an increased molecular understanding of those variants is needed, along with better, more widespread surveillance and control strategies, in order to reduce the impact of this virus.

## 5. Conclusions

It is believed that BRSV is endemic in Turkey; however, minimal information is available on the prevalence of the virus, along with an understanding of the strains present. In order to successfully control infection, defining the target is essential. This study reports the identification and isolation of a new strain of BRSV that has not previously been identified in Turkey. It was associated with animals that died from respiratory distress and the strain shows amino acid differences in the Fusion protein (compared to the only other Turkish isolate) that are known to contribute to virulence. Importantly, the virus has been isolated in culture, which will allow further investigations into its virulence and the significance of those amino acid changes.

## Figures and Tables

**Table 1:** Information on the primers and probes used in the analysis.

Primers and Probe	Sequences (5′-3′)	Product Size(bp)	Ref.
F primer	GCAATGCTGCAGGACTAGGTATAAT	124	Boxus et al. [17]
R primer	ACACTGTAATTGATGACCCCATTCT		
Probe	FAM-ACCAAGACTTGTATGATGCTGCCAAAGCA-TAMRA		
B1	AATCAACATGCAGTGCAGTTAG	711	Vilcek et al. [18]
B2A	TTTGGTCATTCGTTATAGGCAT		
B3	GTGCAGTTAGTAGAGGTTATCTTAGT	481	
B4A	TAGTTCTTTAGATCAAGTACTTTGCT		

**Table 2:** Percentage of nucleotide and amino acid similarity between the isolates identified in this study (marked with *) and other bovine respiratory syncytial virus (BRSV) strains obtained from Genbank. NA: Not available.

	GenBank Number	Strain Name and Country	Amino Acid Similarities (%)												
			NC038272	FJ543091	M82816	D00953	MG947594	KU159366	KY499619	MT179304*	MT024766*	MT024767*	AF092942	AF124561	MF153477
**Nucleotide Similarities (%)**	**NC038272**	ATCC51908 NA		96.05	96.05	97.65	98.44	93.60	92.77	93.60	93.60	93.60	98.44	100.00	95.24
	**FJ543091**	BRSV-25-BR Brasil	96.14		98.44	95.24	96.05	89.38	88.51	89.38	89.38	89.38	94.42	96.05	91.09
	**M82816**	NA NA	96.14	99.49		95.24	96.05	90.24	89.38	90.24	90.24	90.24	94.42	96.05	91.93
	**D00953**	RB 93 NA	98.20	96.40	96.40		97.65	91.93	90.24	91.09	91.09	91.09	96.05	97.65	92.77
	**MG947594**	Lovsta 2016 Sweden	97.69	95.89	95.89	98.46		91.93	91.09	91.93	91.93	91.93	96.85	98.44	93.60
	**KU159366**	USII/S1 USA	96.14	93.06	93.57	94.60	94.09		97.65	98.44	98.44	98.44	93.60	93.60	93.60
	**KY499619**	BRS/TR/Erz/2014 Turkey	95.63	93.06	93.57	94.09	93.57	97.43		99.22	99.22	99.22	92.77	92.77	92.77
	MT179304 *	07TR2019 Turkey	95.63	93.57	94.09	94.09	93.57	97.43	99.49		100.00	100.00	93.60	93.60	93.60
	MT024766 *	34TR2018 Turkey	95.63	93.57	94.09	94.09	93.57	97.43	99.49	100.00		100.00	93.60	93.60	93.60
	MT024767 *	43TR2018 Turkey	95.63	93.57	94.09	94.09	93.57	97.43	99.49	100.00	100.00		93.60	93.60	93.60
	**AF092942**	ATue51908 Germany	99.49	95.89	95.89	97.94	97.43	96.14	95.63	95.63	95.63	95.63		98.44	95.24
	**AF124561**	A2Gelfi France	99.49	95.63	95.63	97.69	97.17	95.63	95.12	95.12	95.12	95.12	98.97		95.24
	**MF153477**	BRSV-UnepJab-1 NA	96.66	93.06	93.06	95.12	94.60	94.86	94.34	94.34	94.34	94.34	96.66	96.14	

**Table 3:** Partial alignment of the F gene from the isolates identified in this study (marked with *), with a selection of strains from the different subgroups. NA: Not available.

Sub- Groups	GenBank Number	Strain Name	Amino Acid Positions of BRSV F Gene Sequence																			
			67	70	71	75	80	91	100	101	102	104	105	113	114	115	118	124	148	168	173	176
I	FJ543091	BRSV-25-BR	N	N	G	K	K	V	E	P	T	S	S	E	S	I	T	K	I	N	S	K
	M82816	NA	.	.	.	.	.	A	.	.	.	.	.	.	.	.	.	.	.	K	.	.
II	D00953	RB 94	.	.	S	N	.	.	.	.	A	.	.	.	L	.	K	.	.	K	.	.
	MG947594	Lovsta 2016	.	.	S	.	.	.	.	L	A	.	.	.	L	.	.	.	.	K	.	.
III	KU159366	USII/S1	D	K	S	.	.	T	V	.	A	F	N	.	L	M	A	R	.	K	.	.
	KY499619	BRS/TR/Erz/2014	D	K	S	.	.	T	V	.	A	F	N	.	L	M	.	R	.	K	T	E
	MT179304 *	07TR2019	D	K	S	.	.	T	V	.	A	F	N	.	L	M	.	R	.	K	T	.
	MT024766 *	34TR2018	D	K	S	.	.	T	V	.	A	F	N	.	L	M	.	R	.	K	T	.
	MT024767 *	43TR2018	D	K	S	.	.	T	V	.	A	F	N	.	L	M	.	R	.	K	T	.
IV	AF092942	ATue51908	.	K	S	.	.	.	.	.	A	F	.	.	L	.	.	.	V	K	.	.
V	AF124561	A2Gelfi	.	.	S	.	.	.	.	.	A	F	.	.	L	.	.	.	.	K	.	.
	NC038272	ATCC51908	.	.	S	.	.	.	.	.	A	F	.	.	L	.	.	.	.	K	.	.
Untyped	MF153477	BRSV-UnepJab-1	.	K	S	.	Q	I	.	.	A	F	.	G	L	T	.	R	.	K	.	.

## Data Availability

All data are available online. Sequences obtained in this study can be found in the NCBI GenBank database at https://www.ncbi.nlm.nih.gov/nucleotide/.
